# Effects of Th17 cells and IL‐17 in the progression of cervical carcinogenesis with high‐risk human papillomavirus infection

**DOI:** 10.1002/cam4.1279

**Published:** 2017-12-26

**Authors:** JiSen Xue, YuLi Wang, Cheng Chen, XueJie Zhu, Hua Zhu, Yan Hu

**Affiliations:** ^1^ The Department of Gynecology The First Affiliated Hospital of Wenzhou Medical University Wenzhou Zhejiang 325000 China; ^2^ The Department of Gynecology The Second Affiliated Hospital of Wenzhou Medical University Wenzhou Zhejiang 325000 China

**Keywords:** Cervical lesions, human papillomavirus, IL‐17, T‐cell immunity, Th17 cells

## Abstract

The existence of Th17 cells and IL‐17 was recently shown in several types of infectious diseases, but their distribution and functions in cervical lesions with high‐risk human papillomavirus (HPV) infection have not been fully elucidated. In this study, the frequency of Th17 cells in peripheral blood samples obtained from 28 cervical squamous cell carcinoma patients, 26 CIN1 patients, 30 CIN2 patients, 29 CIN3 patients, 25 high‐risk HPV‐infected women with normal cervical cytology, and 30 healthy controls was determined by flow cytometry. Besides, the levels of IL‐17 in peripheral blood samples as well as in supernatant of cervical tissue homogenate were assessed by enzyme‐linked immunosorbent assay (ELISA) simultaneously. We found that during the disease progression of cervical lesions, the proportion of Th17 cells in the total CD4^+^ cells showed a gradually increased tendency compared with the controls (*P *<* *0.05). Moreover, levels of IL‐17 in serum and supernatant of cervical tissue homogenate showed the same tendency as the proportion of Th17 cells (*P *<* *0.05). When compared in pairs, the levels of IL‐17 in supernatant differed significantly among the study groups and the control group (*P *<* *0.05), but no significant difference was observed in serum (*P *>* *0.05). In conclusions, the results indicate that Th17 cells and IL‐17 may play a role of immune enhancement in the infection of high‐risk HPV especially in the cervical microenvironment, which contribute to the disease progression of its associated cervical lesions.

## Introduction

The worldwide incidence and mortality of cervical cancer still ranks as the second most common cancer among women worldwide, accounting for nearly 528,000 new cases worldwide and half of them die of cervical cancer 1. About 85% cases occur in the developing countries 1 and China is the second most affected country, about 98,900 new patients in 2015 2. Persistent infection of high‐risk human papillomavirus (HPV) has been established as the necessary and basic cause for developing cervical cancer and its precursor lesions, cervical intraepithelial neoplasia (CIN) 3. HPV infection, in most cases, is self‐limiting and can be eradicated by humoral and cell‐mediated immune response. This suggests that the responses of the host immune systems, especially the cellular immune response, play an important role in the control of both HPV infections and HPV‐associated cervical lesions 4, 5. There is also growing evidence that CD4^+^ T‐helper (Th) cells play an important role in maintaining immune responses against cancer 6, 7. So, T‐cell‐mediated immune responses against oncogenic HPV are believed to play a central role in cervical carcinogenesis. Recently,a distinct subset of CD4^+^ T‐helper (Th) cells characterized by production of interleukin‐17 (IL‐17), now widely known as Th17 cells, has substantially advanced our understanding of the classic CIN Th1/Th2 pattern 8. Recent data suggest that Th17 cells and the mainly effective factor—IL‐17 play an important role in host immune responses to the conditions of inflammation, autoimmunity, and allergic reactions 9, 10, 11, 12, and may be involved in viral infection and the pathogenesis of its associated diseases 13, 14, 15, 16, 17. This study is designed to investigate the percentage of Th17 cells in the total CD4^+^ cells and the expression of IL‐17 in patients with CIN and cervical squamous cell carcinoma (SCC) with high‐risk HPV infection, with the aim to explore the potential role of immunologic response mediated by Th17 cells/IL‐17 during the disease progression and carcinogenesis of (pre)malignant cervical neoplasia which are closely associated with high‐risk HPV infection.

## Materials and Methods

### Ethics statement

Enrollment took place between December 2010 and June 2013 in The First Affiliated Hospital of Wenzhou Medical University. Our research was approved by the Medical Ethical Committee of The First Affiliated Hospital of Wenzhou Medical University. A written informed consent was obtained from each participant. Informed consent declared that remnants of the patient's peripheral blood and cervical tissues excised during surgery would be used for the research.

### Study groups

The study population consisted of 138 subjects, including 28 cervical SCC patients, 26 CIN1 patients, 30 CIN2 patients, 29 CIN3 patients, and 25 high‐risk HPV‐infected women with normal cervical cytology. All subjects enrolled in this study were diagnosed and treated at the Department of Gynecology, The First Affiliated Hospital of Wenzhou Medical University between December 2010 and June 2013. The selection criterion was each participant needed to have a positive result of high‐risk HPV test. Individuals with concurrent autoimmune disease, active or chronic infection, cardiovascular diseases, connective tissue diseases, or a history of malignant tumors were excluded. None of the patients previously received immunosuppressive treatment, radiotherapy, or chemotherapy. The diagnosis of CIN and cervical SCC were confirmed by pathological histology. Both blood and tissue samples were collected before treatment. The characteristics of the studied population are summarized in Tables 1 and 2, and no significant difference were observed (*P *>* *0.05).

**Table 1:** Demographic and clinical characteristics of subjects. Cervical carcinoma stage ≤ IIa means patients diagnosed at early stage (FIGO stage ≤ IIa) can undergo surgery

Stratified factors	Control group	Study group				
		High‐risk HPV infected group	CIN1	CIN2	CIN3	Cervical carcinoma (stage≤IIa)
No. of patients	30	25	26	30	29	28
Age	42.9 ± 10.8	39.5 ± 6.8	39.5 ± 5.8	42.9 ± 9.1	42.2 ± 10.3	48.6 ± 10.7
First‐time sex	21.3 ± 4.5	20.7 ± 2.3	18.9 ± 3.4	21.1 ± 2.8	20.4 ± 3.2	19.3 ± 4.2
Sex partner						
1 (%)	25 (83.3)	20 (80.0)	20 (76.9)	24 (80.0)	22 (75.9)	23 (82.1)
>1 (%)	5 (16.7)	5 (20.0)	6 (23.1)	6 (20.0)	7 (24.1)	5 (17.9)
Gravidity						
1	4	4	2	3	5	3
2	11	8	10	12	10	11
≥3	15	13	14	15	14	14
Parity						
1	11	12	10	13	11	6
2	14	9	12	11	14	13
≥3	5	4	4	6	4	9

**Table 2:** Distribution of high‐risk HPV genotyping among study groups

Type of high‐risk HPV infection	High‐risk HPV infected group	CIN1	CIN2	CIN3	Cervical carcinoma (stage ≤ IIa)
	*N* (%)	*N* (%)	*N* (%)	*N* (%)	*N* (%)
Single infection					
16	5 (20.0)	9 (34.6)	10 (33.3)	13 (44.8)	9 (32.1)
18	3 (12.0)	2 (7.7)	4 (13.3)	1 (3.4)	2 (7.1)
52	2 (8.0)	3 (11.5)	1 (3.3)	2 (6.9)	3 (10.7)
58	4 (16.0)	1 (3.8)	3 (10.0)	2 (6.9)	3 (10.7)
31	1 (4.0)	0 (0.0)	0 (0.0)	1 (3.4)	1 (3.6)
33	1 (4.0)	2 (7.7)	1 (3.3)	2 (6.9)	1 (3.6)
45	0 (0.0)	1 (3.8)	2 (6.7)	0 (0.0)	0 (0.0)
51	0 (0.0)	0 (0.0)	0 (0.0)	0 (0.0)	1 (3.6)
56	0 (0.0)	0 (0.0)	0 (0.0)	0 (0.0)	0 (0.0)
35	0 (0.0)	0 (0.0)	0 (0.0)	1 (3.4)	0 (0.0)
59	0 (0.0)	0 (0.0)	0 (0.0)	0 (0.0)	1 (3.6)
39	1 (4.0)	0 (0.0)	0 (0.0)	1 (3.4)	0 (0.0)
68	1 (4.0)	0 (0.0)	1 (3.3)	0 (0.0)	1 (3.6)
53	0 (0.0)	1 (3.8)	0 (0.0)	0 (0.0)	0 (0.0)
66	0 (0.0)	0 (0.0)	1 (3.3)	0 (0.0)	0 (0.0)
Mixed infection					
16 + 52	2 (8.0)	1 (3.8)	2 (6.7)	2 (6.9)	2 (7.1)
16 + 18	0 (0.0)	0 (0.0)	1 (3.3)	1 (3.4)	1 (3.6)
16 + 33	1 (4.0)	1 (3.8)	0 (0.0)	1 (3.4)	0 (0.0)
16 + 58	1 (4.0)	2 (7.7)	1 (3.3)	0 (0.0)	2 (7.1)
16 + 31	0 (0.0)	1 (3.8)	0 (0.0)	1 (3.4)	1 (3.6)
16 + 45	0 (0.0)	0 (0.0)	1 (3.3)	0 (0.0)	0 (0.0)
16 + 31 + 58	1 (4.0)	0 (0.0)	1 (3.3)	0 (0.0)	0 (0.0)
Other (no type 16)	2 (8.0)	2 (7.7)	1 (3.3)	1 (3.4)	0 (0.0)

### Controls

Thirty patients, who were hospitalized because of uterine myoma were invited to join this study as the control group for providing their peripheral blood samples and cervical tissue samples before the surgery of total hysterectomy. The selection criterion of the control group was that each participant needed to have both normal results of Thinprep cytologic test and high‐risk HPV test.

### Sample preparation

Peripheral whole blood (2 mL) of all groups was collected with coagulator. Next, serum were separated from peripheral whole blood by a centrifugation (2000 r/min for 10 min at room temperature) and stored in −80°C before ELISA analysis. Meanwhile, EDTA‐K2 anticoagulant peripheral whole blood (2 mL) was obtained and stimulated within 4 h for flow cytometric analysis. Cervical tissue biopsy (about 50 mg) was performed in acetowhite and noniodine areas after sterilization. Then, tissue samples were washed thoroughly by cold PBS and stored in liquid nitrogen before homogenation. Equal weights of cervical tissues were homogenized, after which the supernatant was used for ELISA. All samples were collected prior to any treatment.

### Flow cytometric analysis of Th17 cells

To analyze the prevalence of Th17 cells, IL‐17‐producing CD4^+^ cells were evaluated by flow cytometry. In brief, EDTA‐K2 anticoagulant peripheral whole blood (100 *μ*L) with an equal volume of RPMI‐1640 medium was incubated for 4–6 h at 37°C in 5% CO2 in the presence of 25 ng/mL of phorbol myristate acetate (PMA), 250 ng/mL of ionomycin, and 2.5 *μ*g/mL Brefeldin A (all from ENZO, San Diego, CA). Then, the cells were incubated with FITC‐anti Human CD4 monoclonal antibodies (eBioscience, San Diego, CA) at room temperature in the dark for 15 min to stain the surface. After fixation and permeabilization, according to the manufacturer's instructions, the cells were stained with PE‐anti Human IL‐17A and APC‐anti Human IFN‐*γ* in the dark for 15 min. Isotype controls were used with Mouse IgG1 K Isotype Control PE and Mouse IgG1 K Isotype Control APC to enable correct compensation and confirm antibody specificity. The percentage of Th17 cells (IL‐17A^+^IFN‐*γ* ^‐^CD4^+^), Th1 cells (IL‐17A^‐^ IFN‐*γ* ^+^ CD4^+^), and double‐positive cells (IL‐17A^+^IFN‐*γ* ^+^CD4^+^) in the total CD4^+^ cells were detected by flow cytometry using a FACS Calibur cytometer equipped with CellQuest software (BD Bioscience PharMingen).

### ELISA analysis

Serum of all groups were collected and stored in −80°C before analysis. ELISA assay was performed according to the manufacturer's instructions of the ELISA kits. The OD value at 450 nm was measured. The concentrations of IL17 were calculated according to the standard curve. The serum and tissue homogenate concentration of IL‐17 was detected by ELISA analysis for all the study subjects and the control group.

### Statistical analysis

Values were expressed as the Mean ± SD. The data were analyzed by an ANOVA. Comparisons between two groups were assessed by Bonferroni analysis. All tests were performed using the GraphPad Prism 5 system. *P *<* *0.05 was considered statistically significant.

## Results

### Percentages of Th17 cells in peripheral blood showed a gradual elevation during the disease progression of cervical lesions from the controls to the SCC group

**Figure 1:**
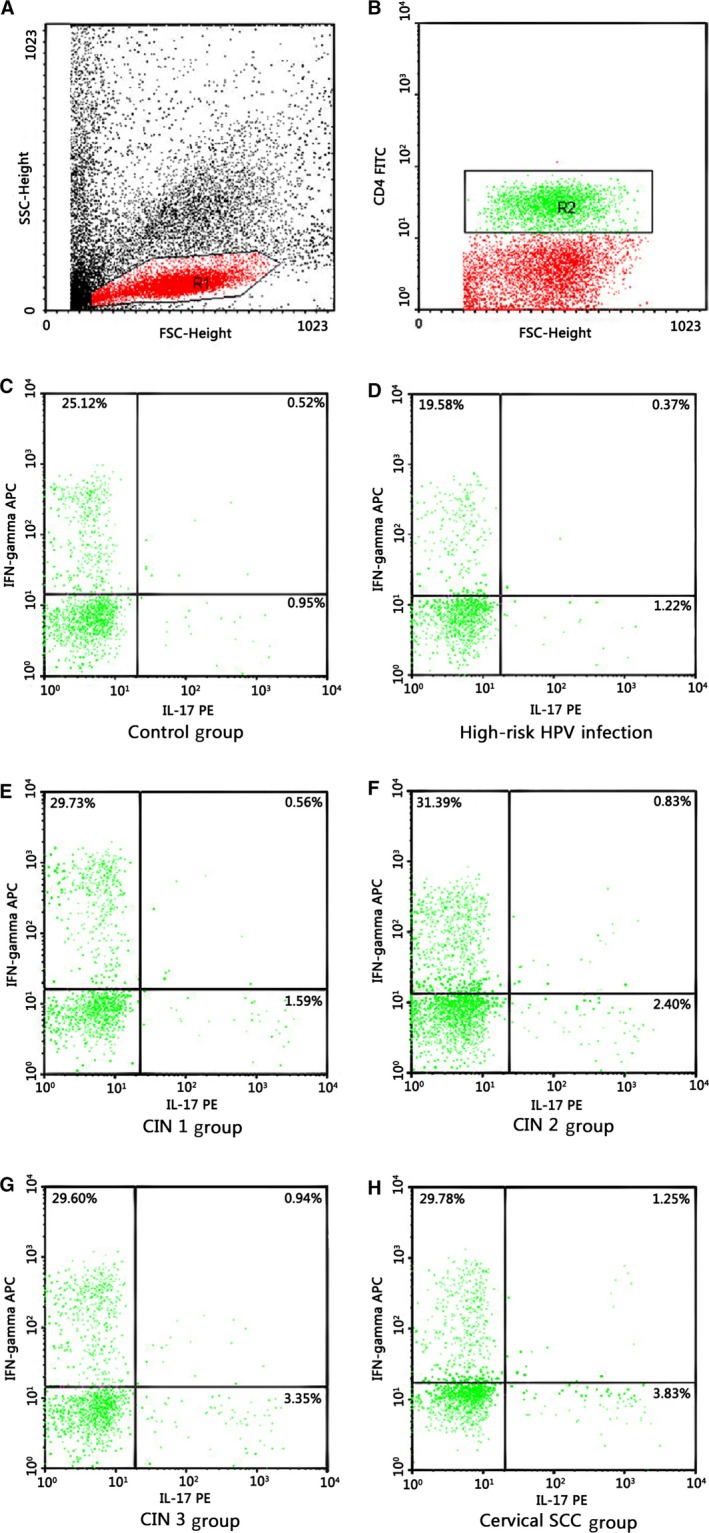
Intracytoplasmic IL‐17 A and IFN‐*γ* expression in peripheral Th cells from individuals of the study groups and the control group were detected by flow cytometry. The cells were stained with FITC‐anti Human CD4, followed by intracytoplasmatically staining with PE‐anti Human IL‐17A and APC‐anti Human IFN‐*γ*. Dot plots show the IL‐17 A and/or IFN‐*γ* secreting CD4^+^T cells. Numbers mean percentages of Th17 cells (IL‐17A^+^ IFN‐*γ* ^‐^ CD4^+^), Th1 cells (IL‐17A^‐^ IFN‐*γ* ^+^ CD4^+^), and double‐positive cells (IL‐17A^+^ IFN‐*γ* ^+^ CD4^+^). (A) Define region 1 (R1) of lymphocytes according to parameters of forward‐angle light scatter and right‐angle light scatter. (B) Define region 2 (R2) of CD4^+^T cells among R1 according to parameter of FITC–CD4 channel. (C–H) Representative of intracytoplasmic IL‐17 A and IFN‐*γ* expression in peripheral Th cells from individuals of the control group and the study groups, respectively.

**Figure 2:**
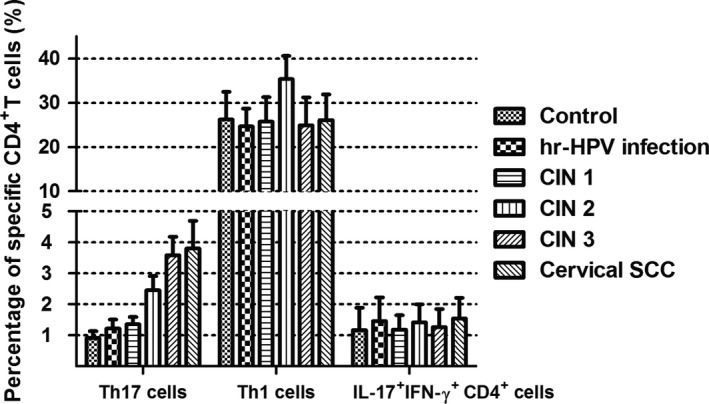
The constituent ratio of specific CD4^+^T cells in peripheral blood samples in the control group and the study groups. After detection by flow cytometry, CD4^+^T cells were divided into three types: Th17 cells (IL‐17A^+^ IFN‐*γ* ^‐^ CD4^+^), Th1 cells (IL‐17A^‐^ IFN‐*γ* ^+^ CD4^+^), and double‐positive cells (IL‐17A^+^ IFN‐*γ* ^+^ CD4^+^).

**Figure 3:**
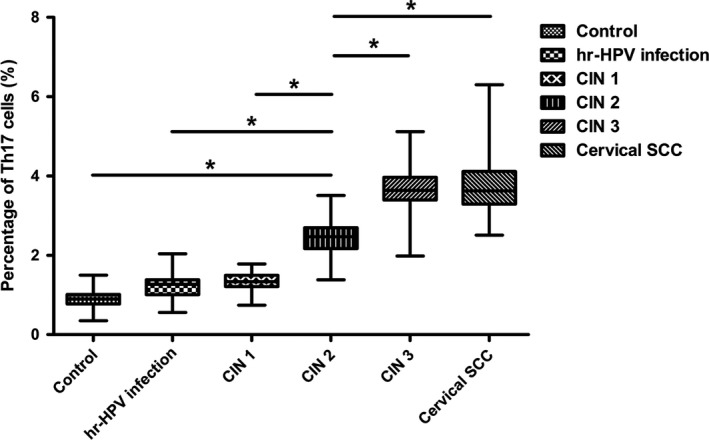
Comparison of Th17 cells (IL‐17A^+^ IFN‐*γ* ^‐^ CD4^+^) ratio in peripheral blood CD4^+^T cells among the control group and the study groups.*P <* *0.05.

After being stimulated with PMA and ionomycin in vitro for a short time, subsets of Th17 cells (IL‐17A^+^IFN‐*γ* ^‐^CD4^+^), Th1 cells (IL‐17A^‐^ IFN‐*γ* ^+^ CD4^+^), and double‐positive cells (IL‐17A^+^IFN‐*γ* ^+^CD4^+^) were detected from peripheral blood samples of all the study subjects and the controls by flow cytometry. The proportions of subsets cells in the total CD4^+^ cells were as follows: Th1 cells > Th17 cells>double‐positive cells (Figs. 1 and 2). Percentages of Th1 cell in CD4^+^ cells were more than 20%, and the double‐positive cells were about 1–2%. Both were not statistically significant among all groups. Notably, percentages of Th17 cells were between 1 and 5%,and during the disease progression of cervical lesions,the proportion of Th17 cells in the total CD4^+^ cells showed a gradually increased tendency compared with the control group (0.905 ± 0.222%). The proportions differed significantly among the study groups and the control group (*P *<* *0.05). The percentages of Th17 cells in the total CD4^+^ cells from peripheral blood samples differed significantly among patients in CIN3 (3.582 ±0.600%)>CIN2 (2.452 ± 0.456%)>CIN1 (1.355 ±0.239%) (*P *<* *0.05). Besides, although the percentage of Th17 cells in the high‐risk HPV infection group (1.217 ± 0.286%) was slightly higher than that in the control group (0.905 ± 0.222%), and was slightly lower than that in the CIN1 group (1.355 ± 0.239%), no significant difference was observed (*P *>* *0.05). Likewise, no significant difference was found between the cervical SCC group (3.801 ± 0.890%) and the CIN3 group (3.582 ± 0.600%) (*P *>* *0.05) (Fig. 3). These results suggest that the immune function of Th17 cells in the cervical cancer patients and the CIN patients was stronger than in the high‐risk HPV infection group and the control group.

### Concentrations of IL‐17 in supernatant of cervical tissue homogenate showed a gradual elevation during the disease progression of cervical lesions as well as in serum

**Figure 4:**
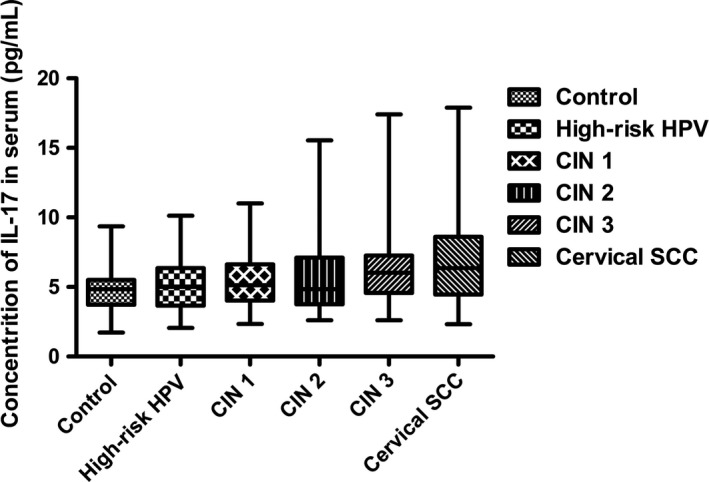
Comparison of IL‐17 levels in serum among the control group and the study groups. After detection by ELISA, IL‐17 levels in patients serum showed a gradually elevated tendency (*P *<* *0.05), but no significant difference was observed when compared in pairs (*P *>* *0.05).

**Figure 5:**
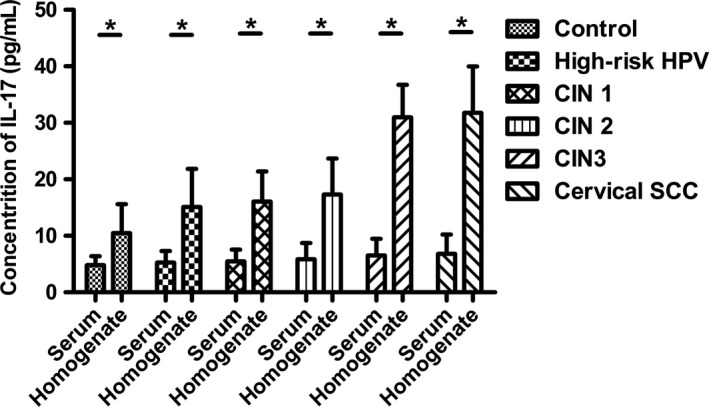
Comparison of IL‐17 levels between cervical tissue homogenate and serum among the control group and the study groups.*P <* *0.05.

**Figure 6:**
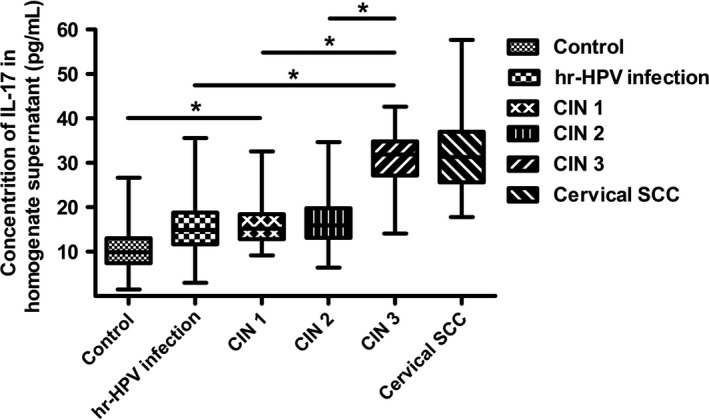
Comparison of IL‐17 levels in supernatant of cervical tissue homogenate among the control group and the study groups.*P <* *0.05.

During the disease progression of cervical lesions, the concentration of IL‐17 in patients serum showed a gradually elevated tendency (*P *<* *0.05), but no significant difference was observed when compared in pairs (*P *>* *0.05) (Fig. 4). Besides, the average concentrations of IL‐17 in supernatant of cervical tissue homogenate of all groups were obviously higher than those in serum (*P *<* *0.05) (Fig. 5). During the disease progression of cervical lesions, levels of IL‐17 in supernatant of cervical tissue homogenate showed a gradually increased tendency which was consistent with the increased prevalence of Th17 cells and IL‐17 in peripheral blood (*P *<* *0.05). The levels of IL‐17 in supernatant differed significantly among the study groups and the control group (10.47 ± 5.125 pg/mL) (*P *<* *0.05) (Fig. 6). When compared in pairs, The levels of IL‐17 from supernatant differed significantly in the control group (10.47 ± 5.125 pg/mL)<CIN1 (16.07 ± 5.333 pg/mL), CIN2 (17.32 ± 6.367 pg/mL)<CIN3 (30.97 ± 5.760 pg/mL) (*P *<* *0.05), but no significant difference was found between the CIN1 group and the CIN2 group (*P *>* *0.05). Besides, although the levels of IL‐17 in the high‐risk HPV infection group (15.11 ± 6.744 pg/mL) was slightly higher than that in the control group (10.47 ± 5.125 pg/mL), and was slightly lower than that in the CIN1 group (16.07 ± 5.333 pg/mL), no significant difference was observed (*P *>* *0.05). Likewise, no significant difference was found between the cervical SCC group (31.79 ± 8.185 pg/mL) and the CIN3 group (30.97 ± 5.760 pg/mL) (*P *>* *0.05) (Fig. 6). Taken together, these data also suggest that the immune function of IL17 in the cervical cancer patients and the CIN patients was stronger than in the high‐risk HPV infection group and the control group, especially in the local cervical tissues.

## Discussion

As we know, T‐helper (Th) cells is a kind of CD4^+^ cell and play an important role in maintaining immune responses, especially the cellular immune response. During the past two decades, some changes in T‐helper cell's classification have been introduced. In 1989 Mosmann and Coffman 18 classified Th cells into two subsets, named Th1 and Th2 cells according to their functional properties and cytokines produced. Th1 cells produce interferon‐*γ* (IFN‐*γ*) and mediate cellular immunity, whereas Th2 cells produce interleukin 4 (IL‐4), IL‐5, and IL‐13 and mediate humoral immunity and allergic responses. This typical immunity response mode was changed by Park 8 and Harrington 19 in 2005 after they discovered a new subset of Th cells that produce IL‐17. This distinct effector lineage of Th cells was named as “Th‐17”.

Since then, lots of studies have indicated that Th17 cells played important roles in several human diseases, including various autoimmune conditions, allergy, the development and progression of tumors, and the acceptance or rejection of transplanted organs and bone marrow inflammation. Focusing on these human studies, here we pay close attention to recent developments regarding Th17 and IL‐17 biology and function in fields of tumors and inflammation by pathogenic microbes infection. In many studies of tumor, Th 17 cells seem to play an active role in antitumor immunity by producing several cytokines like IL‐17 and so on. For example, in patients with advanced ovarian cancer, Th17 lymphocytes accumulate in the tumor tissue and their proportions negatively correlate with disease progression and lead to a significant immune suppression 20. The prevalence of Th17 cells in prostate cancer was negatively correlated with the stage of tumor progression, which also supports a beneficial role for Th17 cells in cancer 21. In lung cancer patients, increased percentage of Th17 cells in malignant pleural effusion predicted longer survival 22. Similar results can also be observed in patients with gastric cancer 23. Meanwhile, in some situations, Th17 cells may hasten tumor development and serve as a detriment to the host. In the study of hormone‐resistant prostate cancer, levels of Th17 cells were inversely correlated with disease progression 24. Increased Th17 cell numbers were detected in colon cancer which suggested that Th17 cells may increase the metastatic ability of tumor cells and accelerate speed of tumor initiation and development 25. So it's still controversy that Th17 cells were friends or foe in patients with cancer. In the study of fungi, Th17 cells were considered to play a predominant role in mediating the CD4^+^ T‐cell response to anti‐C. Albicans host defense 26. The study of C. trachomatis demonstrated that levels of IL‐17 and IL‐22 were high in the cervix of C. trachomatis acutely infected women and suggested that an important fraction of cervical IL‐22‐expressing and IL‐17‐IL‐22‐coexpressing CD4^+^ T cells might play a role in the defense against intracellular pathogens 27. About the function of Th17 and IL‐17 in bacterial infection, there are also several studies. In pulmonary infection, Th17 cell and its effector cytokines, such as IL‐17, IL‐22 and so on, have been proposed to play protective roles 28 and in recent years, Th17 cells have emerged as key players in vaccine‐induced protection against TB 29. In viral infections, the role of Th17 cells is generally considered to be detrimental to the host due to induction of immunopathology. In mouse hepatitis virus (MHV) infection, Th17 cells were found to be responsible for immunopathology in the liver 30 and also been shown to contribute to liver damage in other viral infections, such as HCV 31. Furthermore, in influenza virus infection, Th17 cells markedly increased in the small intestine and lead to diarrhea after PR8 infection, while neutralizing IL‐17A reduced intestinal injury 32. Also, in HSV‐1‐induced corneitis, Th17 cells were shown to enhance immunopathology, synergizing with Th1 cells 33. On the other hand, there are no studies that convincingly show a necessity for Th17 cells to clear a viral infection, but only a few reports elaborate that Th17 cells or their primary cytokine, IL‐17, may be necessary to prevent secondary infections in the HIV infection 34, 35. All these findings suggest that Th17 cells and IL‐17 may also play an important role in the development and progression of cervical carcinogenesis with HPV Infection.

In our study, we analyzed the percentages of Th17 cells in peripheral blood. Our results showed that there were no significant differences in the percentages of Th1 cells and double‐positive cells, while the proportion of Th17 cells showed a gradually increased tendency during the disease progression of cervical lesions compared with the controls (*P *<* *0.05) (Figs. 1 and 2). The percentages of Th17 cells differed significantly among patients with CIN3 > CIN2 > CIN1 (*P *<* *0.05) (Fig. 3). These results indicated an immune enhancement of Th17 cells in cervical lesions with HPV infection. On the other hand, the percentage of Th17 cells in patients with cervical SCC was slightly higher than those in the CIN3 group, but no significant difference was observed (*P *>* *0.05) (Fig. 3). Perhaps this was because all cervical SCC patients enrolled in this study were diagnosed at early stage (FIGO stage ≤ IIa), and the sample size was relatively small. The same situation was discovered in CIN1 versus high‐risk HPV infection group (Fig. 3). Likewise, the possible reason is that because CIN1 is low‐grade neoplasia and most of CIN1 patient can turn to normal. However, concentrations of IL‐17 in supernatant of cervical tissue homogenate of all groups were obviously higher than those in serum (Fig. 5) and significantly differ during the disease progression of cervical lesions (Fig. 6). These results also suggest that IL‐17 might play an immune enhancement role in cervical lesions with HPV infection, especially in the cervical microenvironment.

In summary, we observed significantly gradually elevated levels of Th17 cells and IL‐17 in cervical lesion associated with HPV infection which promotes immune responses. These findings suggest Th17 cells and IL‐17 may be used as a potential clinical examination index and therapeutic target in cervical lesions.

### Ethical approval

The independent Ethics Committee of The First Affiliated Hospital of Wenzhou Medical University approved the study before initiation. All patients gave written informed consent before participation in this study.

## Conflict of Interest

The authors have no financial conflicts of interest.
